# Successful Management of Esophageal Sarcomatoid Carcinoma with Neoadjuvant Chemotherapy and Esophagectomy

**DOI:** 10.70352/scrj.cr.25-0424

**Published:** 2025-09-12

**Authors:** Keiichiro Yokota, Hiroyuki Kitagawa, Kohei Araki, Kento Shinno, Tsutomu Namikawa, Satoru Seo

**Affiliations:** 1 Department of Surgery, Kochi Medical School, Nankoku, Kochi, Japan; 2 Department of Clinical Nursing, Kochi Medical School, Nankoku, Kochi, Japan

**Keywords:** esophageal sarcomatoid carcinoma, neoadjuvant chemotherapy, spindle cell, carcinosarcoma, esophagectomy

## Abstract

**INTRODUCTION:**

Esophageal sarcomatoid carcinoma (ESC) is a rare malignancy characterized by both carcinomatous and sarcomatous components, with no established perioperative chemotherapy standards.

**CASE PRESENTATION:**

We report a case of advanced ESC (T3N1M0, Stage III) treated with 2 courses of neoadjuvant docetaxel, cisplatin, and fluorouracil (DCF) chemotherapy, followed by robotic-assisted thoracoscopic esophagectomy. Histopathology revealed the complete disappearance of spindle cell components, although residual squamous cell carcinoma was found in the flat mucosa. The patient remained recurrence-free for 26 months postoperatively without adjuvant therapy.

**CONCLUSIONS:**

This case suggests that preoperative DCF chemotherapy may be effective for ESC, especially when combined with complete surgical resection, highlighting the importance of addressing both the sarcomatoid and squamous components for long-term survival.

## Abbreviations

CF
: cisplatin and fluorouracil
CK
: cytokeratin
CRT
: chemoradiotherapy
DCF
: docetaxel, cisplatin, and fluorouracil
ESC
: esophageal sarcomatoid carcinoma
FDG-PET
: fluorodeoxyglucose-positron emission tomography

## INTRODUCTION

Esophageal carcinoma is a malignant tumor with a poor prognosis worldwide.^1)^ ESC is a rare malignancy that appears polypoid and consists of mixed carcinomatous and sarcomatous components^2)^ with no established standard treatment for perioperative adjuvant therapy.^3)^ In this report, we present a case of advanced ESC with lymph node metastasis that was successfully treated with preoperative chemotherapy and surgery.

## CASE PRESENTATION

A 69-year-old man was referred to our hospital with suspected esophageal cancer after upper gastrointestinal endoscopy revealed a type I protuberant tumor in the esophagus. The patient had a history of diabetes, hypertension, and dyslipidemia, for which he was taking medications. The patient had also undergone clipping for a cerebral aneurysm.

**Fig. 1:**
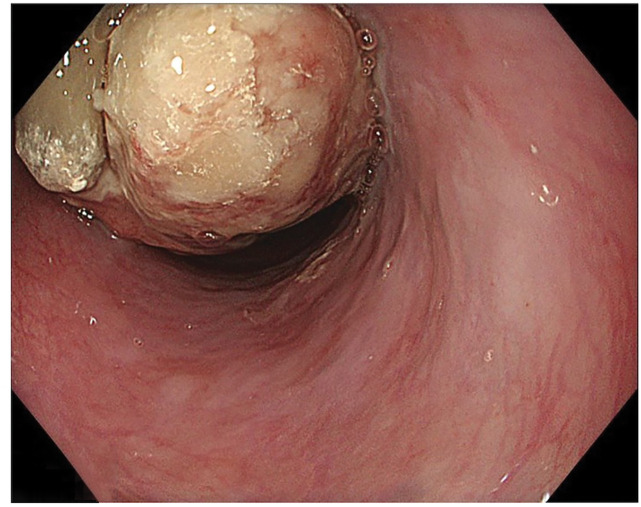
Upper gastrointestinal endoscopy image. A type 1 tumor within the esophageal lumen can be seen on upper gastrointestinal endoscopy.

**Fig. 2:**
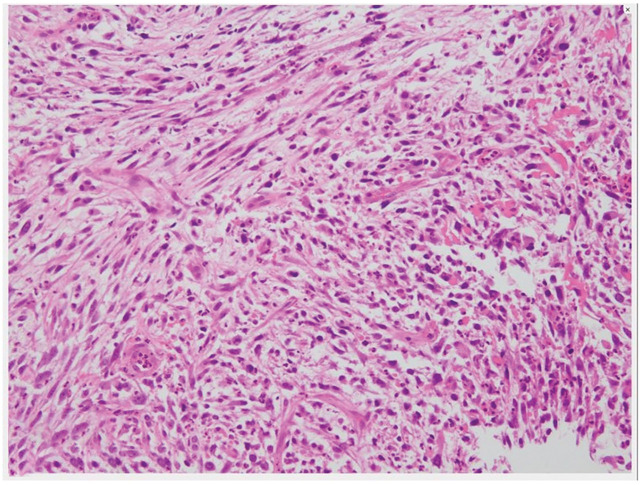
Histopathological examination of the biopsy specimen. The specimen shows proliferation of atypical cells ranging from spindle to rounded shapes.

**Fig. 3:**
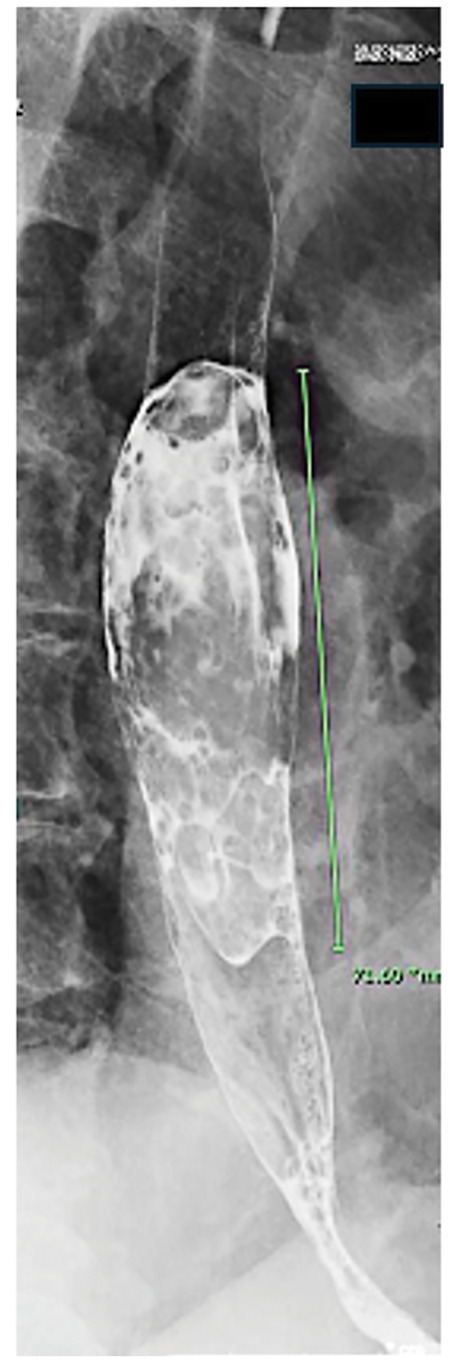
Esophagography image. A mass measuring 70 mm in length in the mid-thoracic esophagus with good barium passage is observed through esophagography.

**Fig. 4:**
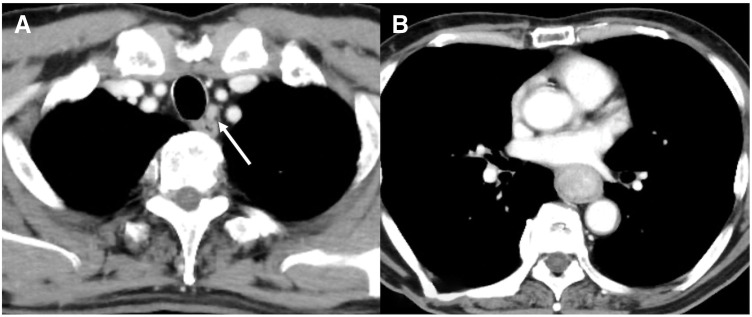
Contrast-enhanced CT image. (**A**) Swelling of the left upper mediastinal lymph node (arrow). (**B**) The tumor bordering the pericardial sac in the mid-thoracic esophagus.

Upper gastrointestinal endoscopy revealed a type 1 esophageal tumor (**Fig. 1**). Biopsy revealed the proliferation of atypical cells ranging from spindle-shaped to rounded (**Fig. 2**). Immunohistochemical staining results were positive for vimentin and negative for CK-AE1/AE3, p40, desmin, CD34, and S100. Esophagography revealed a mass measuring 70 mm in length in the mid-thoracic esophagus with good barium passage (**Fig. 3**). Contrast-enhanced CT revealed a tumor bordering the pericardial sac in the mid-thoracic esophagus and swelling of the left upper mediastinal lymph node (**Fig. 4A** and **4B**). An 18F- FDG-PET-CT revealed increased FDG accumulation in the same area.

**Fig. 5:**
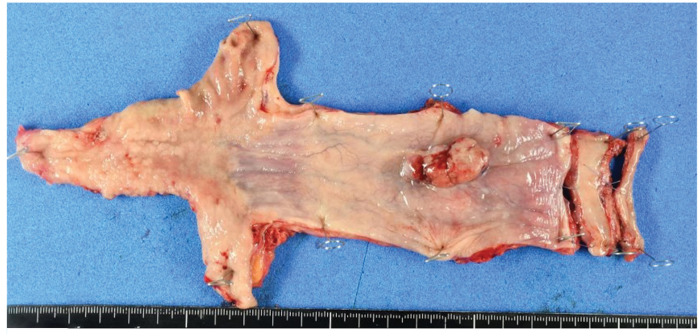
Gross appearance of the esophagectomy specimen. The resected specimen shows a residual stalked mass measuring 30 mm.

**Fig. 6:**
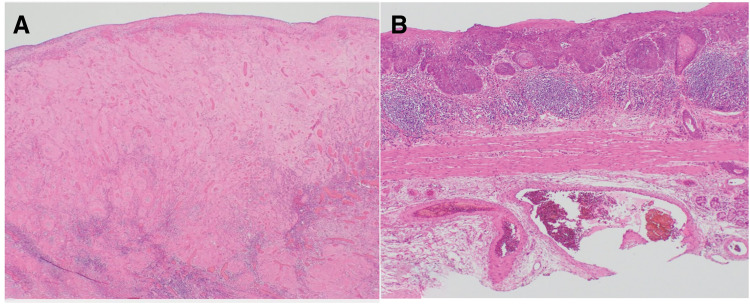
Histopathological examination of the resected specimen. (**A**) The tumor exhibits extensive fibrosis and a dense infiltration of lymphocytes and plasma cells, with no evident spindle cell components. (**B**) Squamous cell carcinoma observed within the flat background epithelium.

Based on these findings, the patient was diagnosed with ESC (T3N1M0, Stage III). Similar to esophageal squamous cell carcinoma, the treatment strategy included 2 courses of DCF (docetaxel, 70 mg/m^2^ on day 1; cisplatin, 70 mg/m^2^ on day 1; and fluorouracil, 700 mg/m^2^ on days 1–5) as preoperative chemotherapy, followed by surgery. A contrast-enhanced CT scan after 2 courses of DCF therapy revealed tumor and lymph node reduction. Subsequently, robot-assisted thoracoscopic esophagectomy and gastric tube reconstruction were performed via the posterior sternal route. The resected specimen revealed a residual stalked mass measuring 30 mm (**Fig. 5**). Pathological examination revealed that spindle tumor cells in the stalked mass had disappeared; however, squamous cell carcinoma was found in some parts of the flat background mucosa (**Fig. 6A** and **6B**). Postoperative anastomotic leakage occurred, but improved with conservative treatment, including bedside drainage and continuous enteral nutrition via a feeding gastro-jejunal tube. The patient was discharged on POD 64. Adjuvant chemotherapy was not performed, and no recurrence was observed 26 months after surgery.

## DISCUSSION

In this case, DCF therapy was administered as preoperative chemotherapy followed by robotic-assisted thoracoscopic esophagectomy for Stage III ESC with lymph node metastasis. The spindle-shaped tumor cells nearly disappeared, and no recurrence was observed for >2 years post-surgery.

As indicated above, ESC is a rare form of esophageal cancer, accounting for only 0.5%–2.8% of all esophageal cancers.^2)^ Pathological examination using preoperative endoscopic biopsy often misidentifies ESC as squamous cell carcinoma.^4)^ To date, standard perioperative chemotherapy for ESC has not been established. Due to its growth into the esophageal lumen, the depth of esophageal wall invasion in ESC is often shallow. Nevertheless, lymph node metastases frequently occur, and its prognosis is poorer than that of superficial esophageal squamous cell carcinoma.^5)^ Therefore, adjuvant chemotherapy during the perioperative period appears to be important for improving survival outcomes. Hashimoto et al. reported 28 ESC patients who underwent surgery between 1990 and 2016, of whom only 2 received preoperative chemotherapy.^6)^ However, recent studies have demonstrated the effectiveness of preoperative chemotherapy for ESC,^7–9)^ consistent with the outcome in our case (**Table 1**). By contrast, postoperative chemotherapy,^10)^ preoperative radiation therapy,^11)^ and preoperative CRT^12)^ for ESC have been reported to result in recurrence within 6–14 months after the surgery. Given these findings, as with stage II or III esophageal squamous cell carcinoma, preoperative adjuvant chemotherapy may be more effective than postoperative adjuvant chemotherapy for ESC.^13)^ Furthermore, CRT for ESCs may result in relapse, even if a temporary reduction is achieved. Therefore, complete surgical resection is preferable for curative treatment. Preoperative DCF therapy for esophageal squamous cell carcinoma showed significantly better survival results than CF therapy.^14)^ Although some reports suggest long-term survival with preoperative CF therapy in ESC, both regimens may be useful.

**Table 1:** Preoperative chemotherapy for esophageal sarcomatoid carcinoma (carcinosarcoma) and outcomes

Author	Age/Sex	Tumor size	Clinical stage	Preoperative chemotherapy	Pathological stage	Postoperative chemotherapy	Outcome
Yoshimoto et al.^7)^	73/Male	50 mm	T3N1M0, Stage III	DCF	T1aN0M0, Stage I	None	No recurrence at 12 months
Ishida et al.^8)^	70/Male	40 mm	T3N1M0, Stage III	CF	T1bN0M0, Stage I	None	No recurrence at 25 months
Kobayashi et al.^9)^	69/Male	60 mm	Not described	CF	T1N0M0, Stage I	None	No recurrence at 5 years
Our case	69/Male	60 mm	T3N1M0, Stage III	DCF	T1aN0M0, Stage I	None	No recurrence at 26 months

CF, cisplatin and fluorouracil; DCF, docetaxel, cisplatin, and fluorouracil

In addition to spindle cell components, ESCs often exhibit squamous cell carcinoma features at the stalk or base.^15)^ Superficial esophageal squamous cell carcinoma is not typically treated with chemotherapy. However, there have been reports of local recurrence after a complete response to DCF therapy, as well as the observation of residual atypical cells during pathological examination of resected specimens.^16,17)^ In the present case, the spindle cell component of ESC resolved with preoperative DCF therapy; however, the coexisting superficial squamous cell carcinoma remained. Therefore, complete tumor resection is essential for long-term survival.

Of course, it is not possible to draw definitive conclusions about the efficacy of preoperative chemotherapy from a single case. More cases need to be studied before any conclusions can be made. Additionally, it is important to recognize the diagnostic challenges specific to ESC and the possibility of sampling bias.

## CONCLUSIONS

We encountered a case in which neoadjuvant chemotherapy with DCF was effective for advanced ESC with lymph node metastasis.
